# Quantifying Infiltration for Quality Control in Printed Mesoscopic Perovskite Solar Cells: A Microscopic Perspective

**DOI:** 10.1021/acsaem.3c03056

**Published:** 2024-02-24

**Authors:** Carys A. Worsley, Thomas O. Dunlop, Sarah-Jane Potts, Rodrigo Garcia-Rodriguez, Rebecca S. Bolton, Matthew L. Davies, Eifion Jewell, Trystan M. Watson

**Affiliations:** Swansea University, Bay Campus, Neath, Skewen SA18EN, Wales

**Keywords:** perovskite, printable, manufacturing, microscopy, analysis, carbon, infiltration

## Abstract

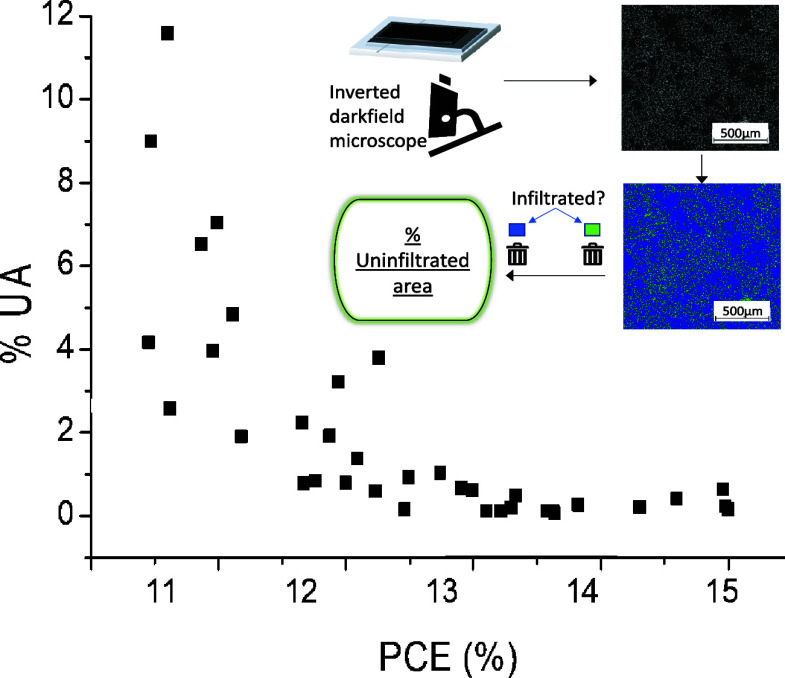

Mesoscopic carbon-based perovskite solar cells (CPSCs) are often cited as a potential frontrunner to perovskite commercialization. Infiltration, the extent to which perovskite fills the mesoporous scaffold, is critical for optimum performance and stability. However, infiltration data are usually presented as qualitative photographic comparisons of samples with extreme infiltration variation. This work examines how small infiltration defects impact performance using an optical microscopy examination of the base TiO_2_ layer to identify issues and develop targeted techniques for infiltration enhancement. Critically, the uninfiltrated area at the base of the stack was found to correlate well with PCE across multiple batches of varied print quality and ZrO_2_ thickness. Through reduction of mesh mark defects and improvement of print quality in the ZrO_2_ and carbon layers, a champion PCE of 15.01% is attained. It follows that this facile, multiscaled, nondestructive technique could enable targeted performance enhancement and quality control in future scale-up initiatives.

## 1 Introduction

Perovskite solar cell (PSC) technologies have undergone drastic development in recent years, with champion PCEs of over 25% surpassing that of some commercially available technologies.^1,1^ However, challenges related to device stability, scale-up, and reproducibility still represent significant barriers to commercial viability.

Mesoscopic printable carbon-based perovskite cells (CPSCs) have been highlighted as a potential frontrunner to commercialization, as they can be fabricated in ambient conditions using easily scaled screen-printing techniques.^2−4^ Consisting of three mesoporous layers of TiO_2_, ZrO_2_, and carbon, which are subsequently infiltrated with perovskite, these devices have been shown to pass stringent IEC61215:2016 stability tests, and modules of 220 cm^2^ active area have been presented in the literature at 9.05% PCE.^5,6^ Concurrent work on scale-up and efficiency is ongoing, with up to 18% PCE now attained for 0.7 cm^2^ devices.^7,8^

As with any PSC architecture, multiple variables exist that impact performance. Perovskite compositions, interfacial materials, band alignment, and physical stack properties all have significant impacts on device performance and stability.^3^ Depending on the chosen perovskite material, optimal stack configuration, thickness, and fabrication environments may vary.

However, there exists a property that must be optimized for peak performance that is universal to all CPSCs regardless of material or architecture selection. Infiltration, the extent to which perovskite fills the mesoscopic stack, is intrinsically linked to performance and stability.^9−12^ Devices with low infiltration experience reduced light absorption and have poor perovskite–electrode contact, producing substandard currents and limited charge extraction.^13^ Poor stack filling has been linked with low crystal quality, increased recombination, and decreased stability.^13−15^

As extreme infiltration issues are largely visible to the naked eye, data are generally presented in the form of qualitative comparisons of samples, most commonly using simple device photographs.^16−18^ However, these necessarily represent only those samples with an extreme infiltration variation. In actuality, samples that appear visually similar can be presented very differently when examined more closely. Although these voids are very small, they have been shown to affect performance.^16^ Performance enhancement due to slight infiltration improvements may therefore be missed, representing a significant potential for erroneous conclusions when comparing device data. Additionally, such problems could render module production commercially unviable, as issues in performance tend to worsen as active area increases.

Although changes in very small voids within the stack may be more closely examined with cross-sectional SEM, this is time-consuming and destructive to the sample, requires specialist equipment, and can be technically difficult. Such an analysis also represents an extremely small portion of the sample, providing information about a limited linear section of the device. As infiltration can vary significantly across micrometers or even centimeters, such images likely do not provide useful and representative data. This method is also inherently unsuitable for module application due to restrictions on sample size and measured area.

Other methods that could be applied to examine infiltration include photoluminescence (PL) or electroluminescence (EL) mapping and light beam induced current (LBIC) measurements. High-resolution PL and EL have been previously applied to examine areas of varied infiltration in CPSCs identified with optical microscopy.^13^ LBIC measurement has also been applied to examine performance changes across the device area.^19^ Although these techniques provide valuable information about comparative perovskite quality across a device area, they cannot necessarily distinguish between areas of low perovskite crystal quality, poor perovskite/electrode contact, and perovskite free infiltration defects without accompanying photographs or microscopy images of the device. Additionally, examining micro- to nanoscale infiltration defects would require advanced setups with high-resolution mapping capabilities, which do not represent a cost-effective way of monitoring infiltration defects at multiple scales.

The following work pairs optical microscopy and image analysis for a quantitative TiO_2_ infiltration comparison. This method is facile, applicable at multiple scales, and nondestructive, allowing fast comparisons of infiltration across the entire tested device area. Through examining collected data,ZrO_2_ and carbon print qualities were identified as key variables impacting infiltration. This enabled the development of targeted infiltration enhancement strategies for improving the CPSC performance. The resultant manufacturing changes enabled a 2% PCE increase and significantly improved reproducibility.

Critically, % uninfiltrated area (%UA) was found to correlate with PCE across multiple batches using different printing regimes. The technique may therefore be suitable for quality control and batch monitoring in future scaled initiatives, where infiltration variation between samples will likely be slight and detailed, nondestructive analysis over large areas is imperative.

## 2 Experimental Section

### 2.1 Materials

Titanium diisopropoxide bis(acetylacetonate) (TAA, 75% in IPA, Sigma-Aldrich), anhydrous 2-propanol (IPA, 99.5%, Sigma-Aldrich), TiO_2_ paste (30NR-D, GreatCell Solar)), ZrO_2_ paste (GreatCell Solar), carbon paste (Gwent Electronic Materials), and terpineol (95%, Sigma-Aldrich) were used as received. Precursor materials PbI_2_ (99%, Sigma-Aldrich), MAI (CH_3_NH_3_I, anhydrous, Dyesol), 5-ammonium valeric acid iodide (5-AVAI, Dyesol), γ-valerolactone (GVL, Sigma-Aldrich), and anhydrous MeOH (99%, Sigma-Aldrich) were used as received.

### 2.2 Device Fabrication

FTO substrates were patterned with a Nb/YVO_4_ laser (532 nm) before cleaning with ∼2% Hellmanex in deionized water, rinsing with acetone and IPA, and drying with N_2_. Substrates were then placed in a Nano plasma system (Diener Electronics), and plasma was cleaned for 5 min in an O_2_ environment. The substrate was heated to 300 °C on a hot plate, and a compact TiO_2_ blocking layer was deposited by spray pyrolysis of 0.2 M titanium di-isopropoxide-bis(acetylacetonate) in IPA.

To form the mesoporous TiO_2_ layer_,_ the titania paste (30NRD) was diluted 1:1 by weight in terpineol, screen printed, and sintered at 550 °C for 30 min after a slow ramp. Next, ZrO_2_ and carbon were printed and annealed at 400 °C for 30 min each.^1^ Unless otherwise specified, layer thicknesses were 600–800 nm, ∼2–2.4 μm, and ∼12–17 μm for TiO_2_, ZrO_2_, and carbon, respectively. All layers were printed and annealed in ambient conditions.

The AVA_0.03_MAPbI_3_ precursors were prepared by dissolving 0.0086 g 5-AVAI, 0.1753 g MAI, and 0.5062 g PbI_2_ in a mixture of 0.9 mL GVL and 0.1 mL MeOH. All precursors solvent mixes were fabricated in an N_2_ glovebox to the specified concentration and stirred at room temperature until dissolved. Once fabricated, precursors were stored in dark ambient conditions (∼18 °C and 30–60% RH).

Devices were cooled to room temperature in ambient conditions (30–50% RH, 18–21 °C) before drop casting of 18–20 μL of room temperature precursor onto the stack surface. Devices were left for 22 min in ambient conditions after drop casting the precursor to ensure adequate infiltration before annealing on a hot plate for 1 h at 50 or 45 °C unless otherwise stated. Contacts were applied with an ultrasonic solder at 190 °C.

### 2.3 IV Testing

The 1 cm^2^ active area was masked to 0.16 cm^2^ for testing. To ensure identical mask placement over multiple tests, tested areas (in the center of the active area) were marked prior to testing. A Keithley 2400 source meter and class AAA solar simulator (Newport Oriel Sol3A) at 1 sun were used for *J–V* measurements (calibrated against a KG5 filtered silicon reference cell, Newport Oriel 91150-KG5). Devices were scanned at a rate of 100 mV s^–1^ from −0.2 to 1.1 V and vice versa after a light soaking period of 180 s. This was performed to account for the well-characterized initial slow response of AVA- containing devices.^20^

### 2.4 Optical Microscopy for Infiltration Comparison

The tested area of each device was marked with a permanent marker before IV testing and optical analysis to ensure that the imaged and tested areas were identical. Images were taken through the glass substrate of completed devices by using a Zeiss Axio Observer ZIM inverted compound microscope. To improve contrast between infiltrated and uninfiltrated areas, dark-field imaging was used. Images were stitched using the Zeiss control software, which was then analyzed in the Zeiss ZEN Blue software.

For quantitative image analysis, images were brightness and contrast equalized before a contrast lookup table was applied to maximize the contrast between infiltrated and uninfiltrated areas (UAs) and remove any glass reflections. Pixels were then binned according to color, and resultant data were used to calculate %UA. Machine automated image segmentation, trained on a large number of carbon cells using the Zeiss Intellesis framework, was used to calculate %UA.

### 2.5 Electroluminescence Measurements

EL was performed on fully fabricated unencapsulated devices after a 7 day settling period to attain peak performance. An FS5 Spectrofluorometer (Edinburgh Instruments) with a Keithley 2401 Source Meter Unit was used for all measurements. All samples were measured under a 3 V applied bias to obtain high emission. Excitation and emission bandwidths were 0 and 3 nm, respectively, with a neutral density filter of O.D. 5 in the excitation pathway. A 700–850 nm range was used with a step size of 0.25 nm and 0.1 s dwell time. Images were obtained every 30 s after bias application to monitor EL evolution. For current samples, a constant device current of 0.1 A was maintained for 30 s before the EL was measured.

### 2.6 White Light Interferometry

White light interferometry was performed on ZrO_2_ layers printed on FTO with the specified regime. Layers were annealed for 30 min at 400 °C after a slow ramp and cooled to room temperature before white light measurements.

Five-times magnification was used, giving a measurement area of 1.2 by 0.93 mm (at a resolution of 736 × 480 pixels with sampling at 1.67 μm intervals). Average surface roughness measurements (*S*_a_ and *S*_z_) over the printed area were taken from the edges. A total of nine measurements were taken for each setting.

### 2.7 Carbon Sheet resistance

Layers of size 10 × 10 cm^2^ were screen printed using a standard 61–64 carbon mesh onto plain glass. Samples were dried at 150 °C for 30 min and cooled to room temperature before testing. Layers were not annealed for this test as the annealed layers were too mechanically fragile to accurately measure the resistance.

Sheet resistance was measured using an SDKR-13 four-point probe (NAGY Messsysteme GmbH) with a tip distance of 1.3 mm and Keithley 2400 source meter. All measurements were taken from the center of the area. Averages of four measurements were obtained.

### 2.8 Cross-Sectional Images

Samples were scribed on the glass substrate and snapped before subsequent broad beam ion milling for 1.5 h at 4 kV in a Hitachi IM4000 broad beam argon ion miller (Hitachi, Tokyo, Japan) with an argon flow of 0.07 cm^3^/min according to a previously established work.^18^ After mounting for examination, samples were observed using an optical microscope with 100× magnification and a polarizing lens rotated to highlight graphite flakes aligned horizontally to the zirconia layer. Images were obtained using an attached iPad tablet.

### 2.9 Printing Imaging

High-speed imaging of the print cycle on the apparatus was conducted at the interface between the screen and the substrate using a Photron FastCam Mini High-Speed Camera (Photron, Tokyo, Japan) at a frame rate of 125 frames per second with 5× magnification, and a 10,000 lx lamp was used for backlighting. Camera images were processed in ImageJ (Version. 1.52v). For each test, the lengths of these flow regions were measured over 15 evenly spread intervals across the visible length of the print process, where the full contact region (where the ink was in simultaneous contact with the mesh and substrate) could be seen (image frames taken from around every 0.024 s). Tests were done on carbon pastes of 0, 5, and 10 wt % % dilution with 1-methoxy-2-propanol. Each paste was printed three times, with each print assessed using ImageJ as described previously, producing 45 measurements that were used to calculate the average lengths of the flow regions for each dilution.

### 2.10 Electrochemical Impedance Measurements

Measurements were performed on unmasked devices using a Zahner CIMPS-X photoelectrochemical workstation. Measurements were performed over the frequency range of 10 MHz to 1 Hz at open circuit under illumination from a red LED (630 nm) with 1 Sun equivalent intensity.

### 2.12 Profilometry

Thickness measurements and surface roughness values were obtained using a Dektak D150 profilometer with a 12.5 μm stylus diameter and force of 3 mg. Samples were measured across the whole printed area, and step heights were obtained for both edges of the print when possible. All presented average values include data from three or more such measurements.

## 3 Results and Discussion

### 3.1 The Impact of Infiltration

**Figure 1:**
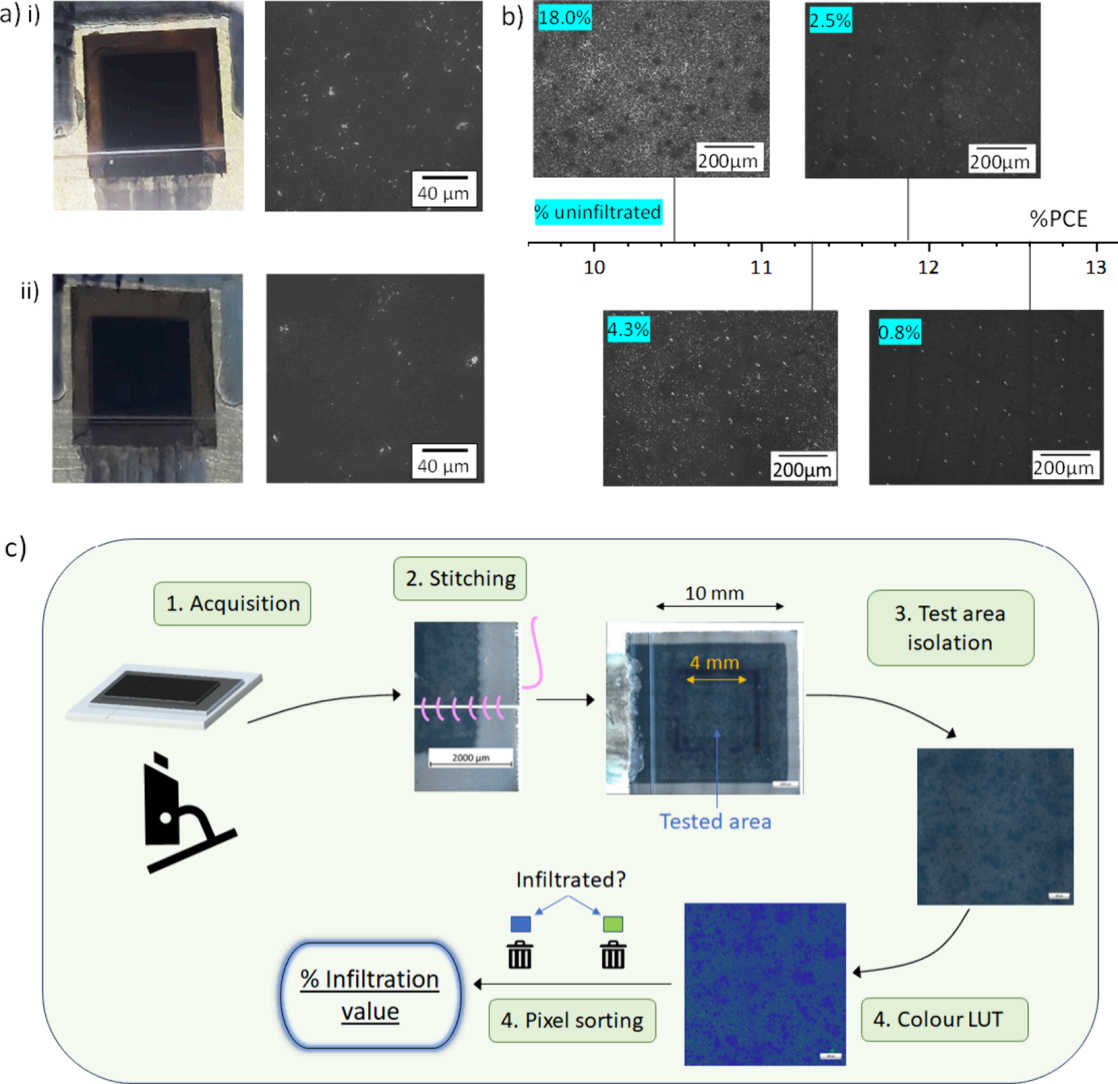
(a) Visually similar devices with infiltration differences visible upon examination with a microscope. (b) Example optical images of devices of varied performance from the same batch. Optical microscopy images are magnified for clarity and thus do not represent the whole tested area in this instance. (c) Diagrammatic representation of the method used for calculating % uninfiltrated area (%UA).

Optical microscopy can reveal infiltration differences between two visually similar devices. This can be seen clearly in Figure 1a, which shows that although appearing identical in photographic images, the two devices show differences under closer microscopic scrutiny. As uninfiltrated areas present as light against darker perovskite-filled sections, it was hypothesized that the percentage of uninfiltrated TiO_2_ area (%UA) may be easily calculable through image analysis. Optical microscopy was therefore used to examine devices from a single device batch that showed a varied performance (Figure 1b). The whole tested area (0.16 cm^2^ at the device center) was examined, and obtained images were stitched. The image was colored to better differentiate light and dark pixels before pixel binning to obtain the final %UA value (Figure 1c).

Figure 1b shows optical microscopy infiltration of devices from a batch with mixed device performance with %UA highlighted in blue. This examination revealed a clear difference between the base TiO_2_ filling of the devices (Figure 1b). Remarkably, even a device with 18% UA achieved 10.44% PCE. This highlights the resilience of CPSCs toward changes in perovskite coverage, as the classic sandwich with such poor coverage would likely suffer much more drastically reduced performance.^21^

**Figure 2:**
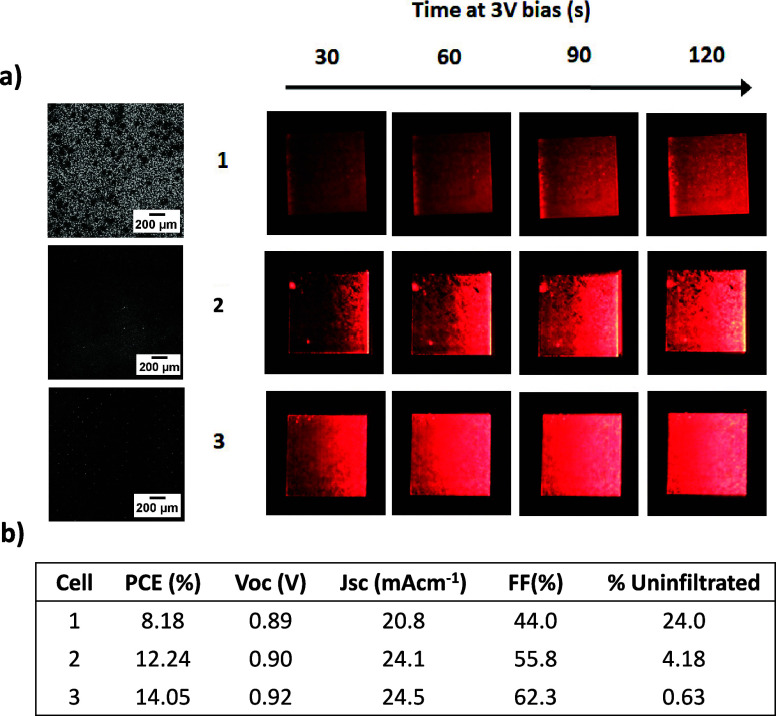
(a) Optical images of the tested area (black) and electroluminescence evolution (red) in three devices of varied infiltration. Optical images depict a small, magnified section for the sake of clarity. (b) PV parameters and %UA (percentage uninfiltrated area) of the three devices.

To examine the impact of infiltration more thoroughly, electroluminescence (EL) measurements were performed on three devices with different %UA values and performance (Figure 2a). Despite the relatively large %UA in samples 1 and 2, surprisingly, homogeneous emission was observed in all three cells (Figure 2a). This may be due to luminescence from the perovskite deeper in the ZrO_2_ layer. Alternatively, observed signals for bare TiO_2_ could be reflecting EL from the surrounding perovskite.^16^ The device with the lowest %UA and highest performance (sample 3) also exhibited the brightest EL, which reduced in intensity as %UA increased for each sample. This indicates that poor infiltration increases levels of nonradiative recombination.^22^

Sample 3 also exhibited faster EL evolution, achieving a bright emission by 30 s and maximum emission by 60 s, whereas sample 1 changed throughout without reaching significant intensity. It should be noted that all samples showed a brighter emission near the carbon electrode during initial measurements. This could be a consequence of charge accumulation and thus faster trap filling at the relatively resistive and poorly selective carbon contact.

Faster EL evolution to brighter intensity is indicative of decreased nonradiative recombination and indicates that crystal quality improves as %UA decreases.^22^ Additionally, faster response times and improved FF indicate that interfacial charge transfer is significantly impacted by %UA.

Electrochemical impedance spectroscopy was therefore performed to more closely examine the impact of infiltration on the charge transfer and recombination.

**Figure 3:**
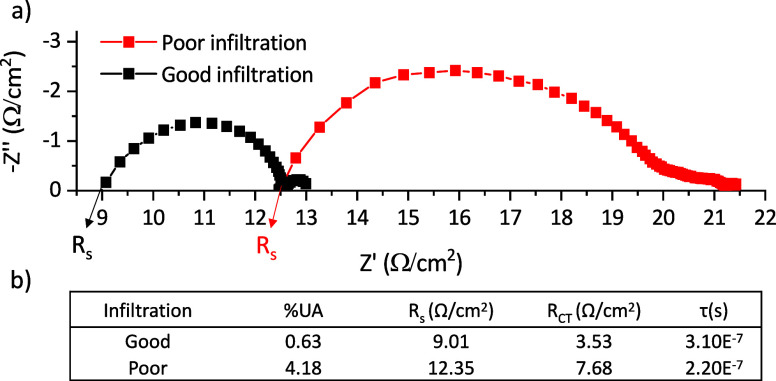
(a) Nyquist plots of high and low %UA cells. (b) Table showing relevant %UA; device; and *R*_s_, *R*_ct_, and τ values calculated from the EIS data.

Figure 3a shows the impedance response of high- and low-%UA devices. A large arc from the high to intermediate frequency region is evident in both devices, with additional features toward the low-frequency region. Although several different electrical equivalent circuit models have been proposed for CPSCs, little consensus exists on the detailed interpretation of EIS data.^23^ In particular, there remains little agreement on the interpretation of the intermediate- and low-frequency regions, the presentation of which varies significantly in the published literature.^24−26^

There is however a broad agreement that the highest frequency response is associated with electrode series resistance and the carbon/perovskite interface (*R*_s_), whereas that of the initial intermediate frequency region is impacted by the TiO_2_/perovskite interface and internal perovskite charge dynamics.^23,24,27^ As such, shifts in the position and diameter of this arc (charge transfer resistance) have been associated with changes in interfacial charge transfer.^24,26^

The first arc in these data is larger and shifted to a higher series resistance in the high-%UA device, resulting in a concurrent drop in calculated electron lifetime (τ) from 3.1 to 2.2E^–7^ (calculated using a semicircle fit). The arc is also significantly wider in the high-%UA cell, with the intermediate-frequency portion at much higher resistance. This suggests that either the perovskite/TiO_2_ interface is more significantly impacted than the carbon or that the perovskite quality is significantly poorer in this sample. In either case, these data confirm that higher %UA increases series resistance within the device, corroborating the previous EL data.

These data agree with previous works, which showed that small uninfiltrated areas reduce charge transfer and act as areas of high recombination.^17^ It appears that larger perovskite free areas have a similar effect. Any proposed method for infiltration monitoring should therefore be suitable for examining defects at different scales across a large area.

The %UA clearly impacts the device performance. It was however unclear whether UA and PCE would significantly correlate across multiple batches, as multiple other factors such as layer thickness, deep stack filling, or graphite flake alignment can affect performance.^16,28−30^

**Figure 4:**
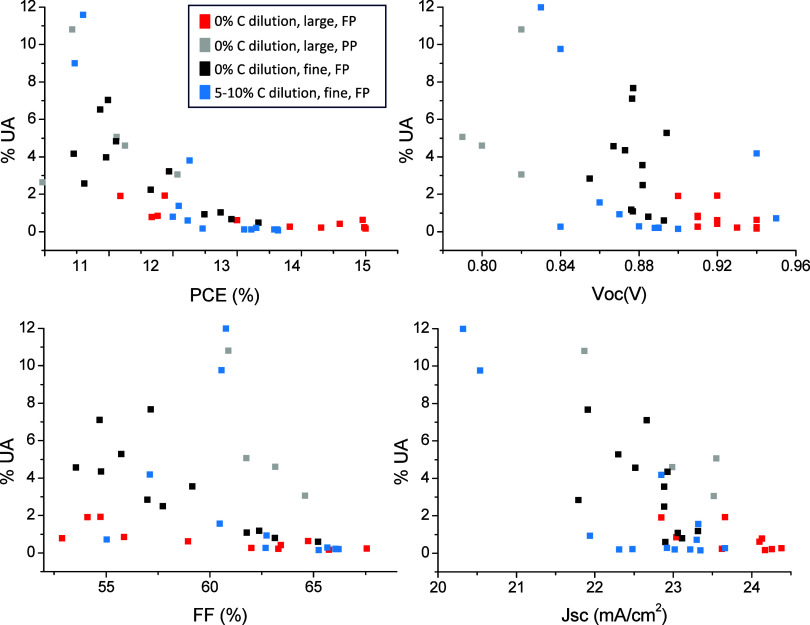
Relationship between % uninfiltrated area (%UA) and device performance. This plot contains data of devices from every batch presented throughout this manuscript, identified by color. Variations include using fine (130–34) vs large (90–48) ZrO_2_ mesh size, diluting the carbon ink (by 0, 5, 10%), and two ZrO_2_ printing regimes. PP refers to a two-pass print regime, and FP refers to a single-pass flood print. The specific impact of each variation is discussed in Section 3.2.

Performance and %UA data from multiple batches with slight manufacturing variations were therefore compiled into a larger plot (Figure 4). The materials used, perovskite formulation, and device composition are identical in all cases. Batches were varied by diluting the carbon ink (by 0, 5, 10%), using fine (130–34) vs large (90–48) ZrO_2_ printing meshes, and trialing two ZrO_2_ printing regimes. PP refers to a two-pass print regime, where paste is deposited through the screen twice with squeegees, and FP refers to a single-pass flood print, where ink is first coated across the screen before a single squeegee pass to deposit the ink. The specific impact of each variation is discussed in Section 3.2.

As shown in Figure 4, plotting % UA area against PCE reveals a clear relationship. As %UA decreases, PCE initially increases sharply up to 12.5% (∼3% UA), after which the rate of improvement is less marked. This is due to general improvements in all photovoltaic parameters with increasing %UA. This result indicates that optical microscopy and paired image analysis could potentially be used for PCE prediction, which would be useful for batch monitoring and quality control in large-scale manufacture. It is important to note that this trendline may shift depending on the chosen perovskite composition: for example, the PCE at each %UA may be higher for FAPbI_3_ devices due to a more optimal perovskite band gap. This work examines cells made by using AVA_0.03_MAPbI_3_. To apply this method for PCE prediction in CPSCs using a different perovskite, a PCE vs %UA calibration curve for the chosen perovskite composition would be required.

Greater variance is present as the %UA increases. This may be due to variations in infiltration pattern: a sample with small, dispersed crystals throughout the TiO_2_ can provide the same overall uninfiltrated area as one with larger, interconnected grains confined to one section. However, the performance may be quite different in such samples: Small, isolated crystals likely have a greater impact on charge transport and recombination due to poor interconnectivity and increased surface area. Expanding the sample set or refining the model to account for crystal spread may allow improved PCE predictions at a higher percentage uninfiltrated area.

The appearance of a common trend among different batches is extremely interesting. Variables such as ZrO_2_ thickness, graphite alignment, and carbon thickness have all been shown to affect PCE and are likely to vary between manually printed batches, particularly where differing print regimes are used.^18,30−32^ It was therefore expected that samples from different batches would produce distinct separate trendlines, for example, samples with thicker ZrO_2_ producing higher PCEs at given low levels of infiltration.

Instead, the alignment of most samples with the general trend suggests that TiO_2_ infiltration quality is the major predictor of performance here. It is possible that ZrO_2_ thickness and other variables become important contributors to PCE once high-quality filling is achieved, producing different trends in different samples with alike infiltration, although improved alignment with the trend at high infiltration % implies that this may not occur. In this case, these data suggest that for a given variable (e.g., interlayer thickness or perovskite formulation), infiltration should first be optimized before representative comparisons can be made. Alternatively, the sample differences in these sets may simply be minor enough not to induce significant deviation from the trend. A systematic comparison of %UA and performance of multiple batches with large ZrO_2_ or carbon thickness variations or different perovskite compositions provides a good opportunity for future work.

### 3.2 Determining the Causes of Poor Infiltration

**Figure 5:**
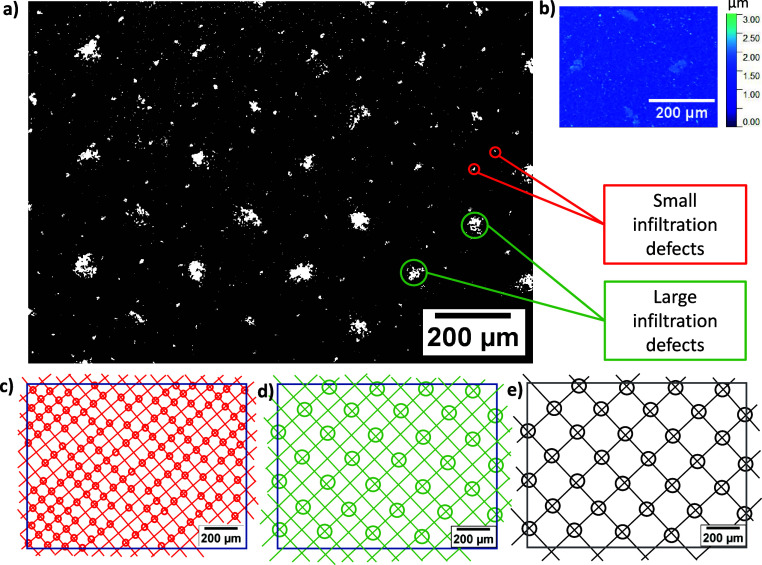
(a) Optical image of infiltrated TiO_2_ with examples of spaced defects circled. (b) White light interferometry (WLI) of mesh marks observed on printed, uninfiltrated 1 L ZrO_2_ films. Panels c, d, and e show diagrams of mesh dimensions of TiO_2_, ZrO_2_, and carbon screens, respectively. The circles represent cross sections where an infiltration issue is present on the optical image.

Upon inspection of the optical images, it was noted that many otherwise well-infiltrated samples exhibited sets of evenly spaced defects. Figure 5a shows a device where two sizes of defects are present at different spacings: the smaller set identified in red and the larger set in green. The smaller defects were less severe and represent a relatively small overall %UA (<1%). However, the larger set produced a sum uninfiltrated area of ∼6.8% (calculated by image analysis). As discussed in Section 3.1, Figure 4, this could significantly impact PCE.

These defects are likely a consequence of mesh marking, characteristic spaced peaks or troughs left by the screen mesh on a printed film when ink remains in contact with mesh crossover points as the screen departs the substrate, forming thin strands of ink (termed “filamentation”). Figure S1 shows a labeled image obtained during screen printing, where filamentation and resultant mesh marks can be seen. It was theorized that the large increases in local surface roughness caused by mesh marking could impact precursor wetting and percolation in these areas, producing the spaced infiltration defects present in Figure 5a.^29,30^

Indeed, the smaller set of defects, identified in red, corresponds to the dimensions of the 130–34 TiO_2_ mesh cross sections (Figure 5c) and are likely therefore due to mesh marking in the TiO_2_ layer. Interestingly, these smaller marks were more common and severe at alternate mesh crossover points. This made identifying the cause of the larger set more difficult, as the spacings correspond to the carbon mesh (Figure 5e) but also to that of alternate ZrO_2_ crossover points (Figure 5d).

As shown by inlaid white light interferometry (WLI) data (Figure 5b), similar marks were observed at the surface of printed ZrO_2_ layers, suggesting that the large defects may be due to marking in this layer.

**Figure 6:**
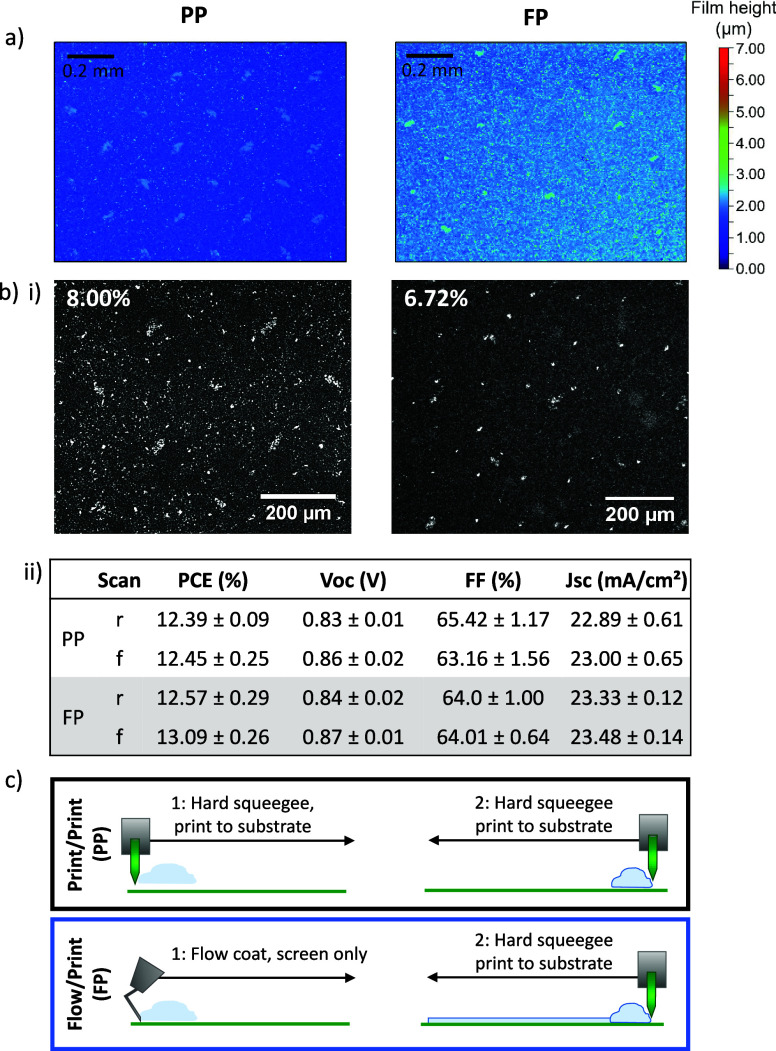
(a) White light Interferometry of ZrO_2_ films on FTO/glass substrates deposited with PP and FP-H printing regimes. (b) (i) Images of infiltrated devices with labeled uninfiltrated area. (ii) Table showing device performance. (c) Diagrammatic representation of PP and FP printing regimes.

These ZrO_2_ layers were deposited with two high-tension squeegee print passes (PP, Figure 6c). This can exacerbate mesh marking by increasing contact time with the underlying paste, providing opportunity for increased ink filamentation.^33,34^ A flow coating print method was therefore trialed to reduce marking, where ink is first spread across the screen before a single print pass (FP, Figure 6c).

The WLI of the produced films is presented in Figure 6a, where it is clear that FP ZrO_2_ layers had fewer, less severe mesh markings than PP films. This resulted in fewer, smaller infiltration defects and a 1.28% reduction in %UA, as presented in Figure 6bi. Consequently, increased *J*_sc_ reproducibility and 0.55% average PCE enhancement were observed in complete devices (Figure 6bii), Figure S2). FP was therefore adopted as the standard method for ZrO_2_ deposition: all devices presented henceforth were prepared in this way.

Although smaller than in previous PP devices, the large defects are still visible in the FP samples (Figure 6bi). Mesh marks become smaller and less severe as thread diameter decreases, as contact between the screen and printed film is minimized. The ZrO_2_ layers presented in Figure 6 were deposited using a 90–48 large mesh, meaning that each mesh was composed of 90 threads per centimeter, each of 48 nm in diameter. Therefore, to further reduce the severity of marks, a fine mesh (130–34) was trialed with the FP method. As finer meshes also allow less ink to pass through the screen, single-layer prints were measured at 1.1 ± 0.1 μm using profilometry (Table S1). To maintain an adequate interlayer thickness, two layers were deposited for cell fabrication.

**Figure 7:**
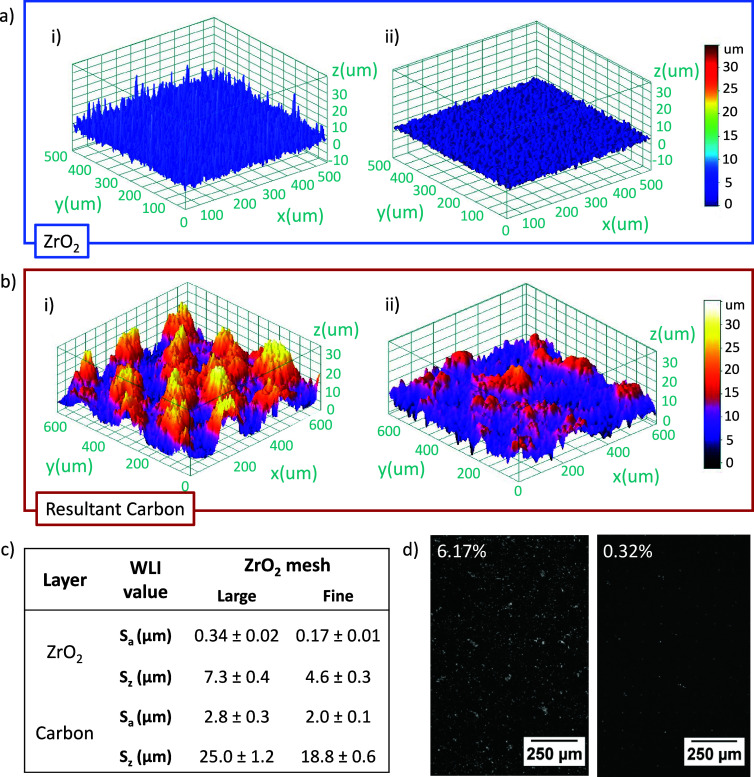
White light interferometry data showing impact of large (i) and fine (ii) ZrO_2_ mesh size on (a) ZrO_2_ and (b) carbon prints. (c) Average roughness (*S*_a_) and average maximum roughness (*S*_*z*_) values. (d) Infiltrated TiO_2_ in completed large mesh and fine mesh devices with % uninfiltrated labeled.

Figure 7a shows WLI images of the resultant ZrO_2_ print surface. The finer mesh produced a much smoother layer, with an average *S*_a_ (arithmetic mean surface roughness) of 171.31 ± 6.06 nm compared to 341.44 ± 24.22 nm for the large mesh (Figure 7c). This was despite a lack of obvious mesh marking in either case, suggesting that fine meshes also improve the bulk print quality. Interestingly, this relatively small change in ZrO_2_ roughness significantly impacted that of the subsequent carbon print (Figure 7b), producing severe mesh marks of 15 μm in height. Large infiltration defects matching the spread and dimensions of these marks were then observed in the large mesh cells (Figure 7d) but were not present in the fine mesh samples. It is therefore likely that the infiltration defects in Figures 3, 6, and 7d were a consequence of carbon mesh marks caused by the increased general roughness in the underlying ZrO_2_.

Resultant device infiltration reflected the observed differences in print quality: the fine mesh samples obtained %UA as low as 0.32% and PCEs up to 14%, whereas those produced with the large mesh exhibited the best %UA of 6.17%. Full box plots are available in Figure S3. However, this was not consistent across different batches, with many devices exhibiting >12% uninfiltrated area even for the fine mesh samples (Figure S4). Consequently, variations of 11–14% PCE were observed.

This may be due to slight changes in ZrO_2_ layer quality between batches, which were shown to drastically affect the carbon print in Figure 7. As printing, slumping, and annealing are all performed in ambient conditions, small topological ZrO_2_ variations may be difficult to avoid. Reducing the sensitivity of the carbon ink toward ZrO_2_ changes might therefore best improve the reproducibility.

**Figure 8:**
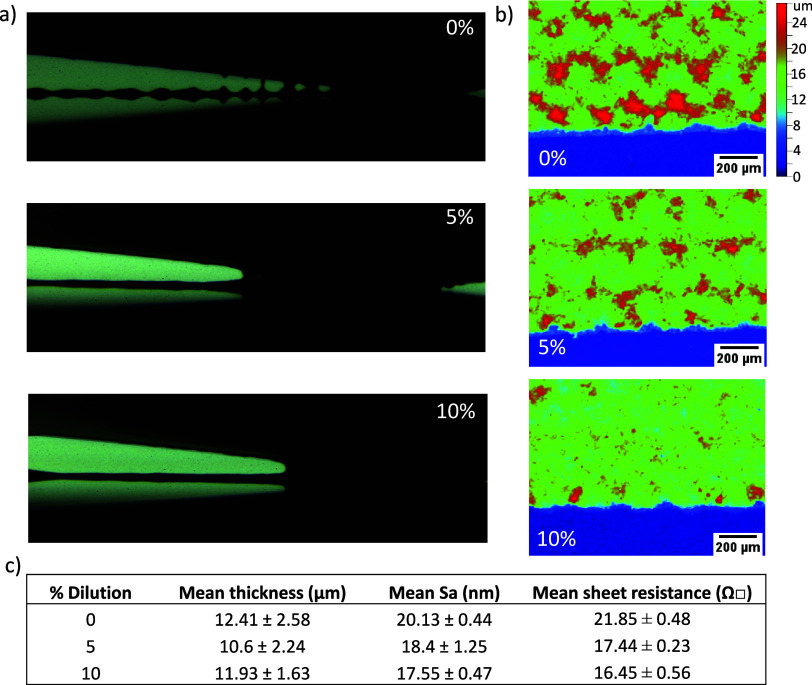
(a) Images showing ink and screen separation of 0, 5, and 10% diluted inks during printing. (b) White light interferometry (WLI) images of stacks prepared with the diluted inks (blue = ZrO_2_, red/green = carbon). (c) Mean WLI thickness and arithmetic mean roughness (*S*_a_) values of diluted carbon inks on ZrO_2_ and average sheet resistance of each ink on glass.

Previous work has shown that dilution with 5–10% 1-methoxy-2-propanol can reduce mesh marking and overall roughness in carbon paste by enabling more effective ink separation from the screen.^33^ Inks with 0, 5, and 10% dilutions were therefore prepared and examined during printing for filamentation changes (Figure 8a). WLI was then applied to examine the resultant carbon electrodes (Figure 8b).

The diluted pastes showed reduced filamentation during printing, resulting in visibly smoother top electrodes with a clear reduction in roughness and mesh marking (Figure 8b). Representing the difference between the maximum peak and trough heights, the average *S*_z_ dropped from 2.16 ± 0.08 μm with the standard paste to 1.78 ± 0.07 and 1.67 ± 0.06 μm for the 5 and 10% dilutions, respectively. Mesh mark severity has therefore fallen with dilution. The *S*_a_, or arithmetic mean roughness, values also fell similarly (Figure 8c), indicating that the overall film quality is improved.

Ink dilution can produce thinner prints, which tend to have increased sheet resistance. However, sheet resistance actually decreased significantly with dilution, with 5 and 10% solvent additions causing a >20% reduction (Figure 8c). This is likely a consequence of decreased roughness: very rough layers have thin areas of low conductivity and thus increased resistivity.^29^ Even without improved infiltration, ink dilution may therefore improve *J*_sc_ and FF due to the increased conductivity of the top electrode.

**Figure 9:**
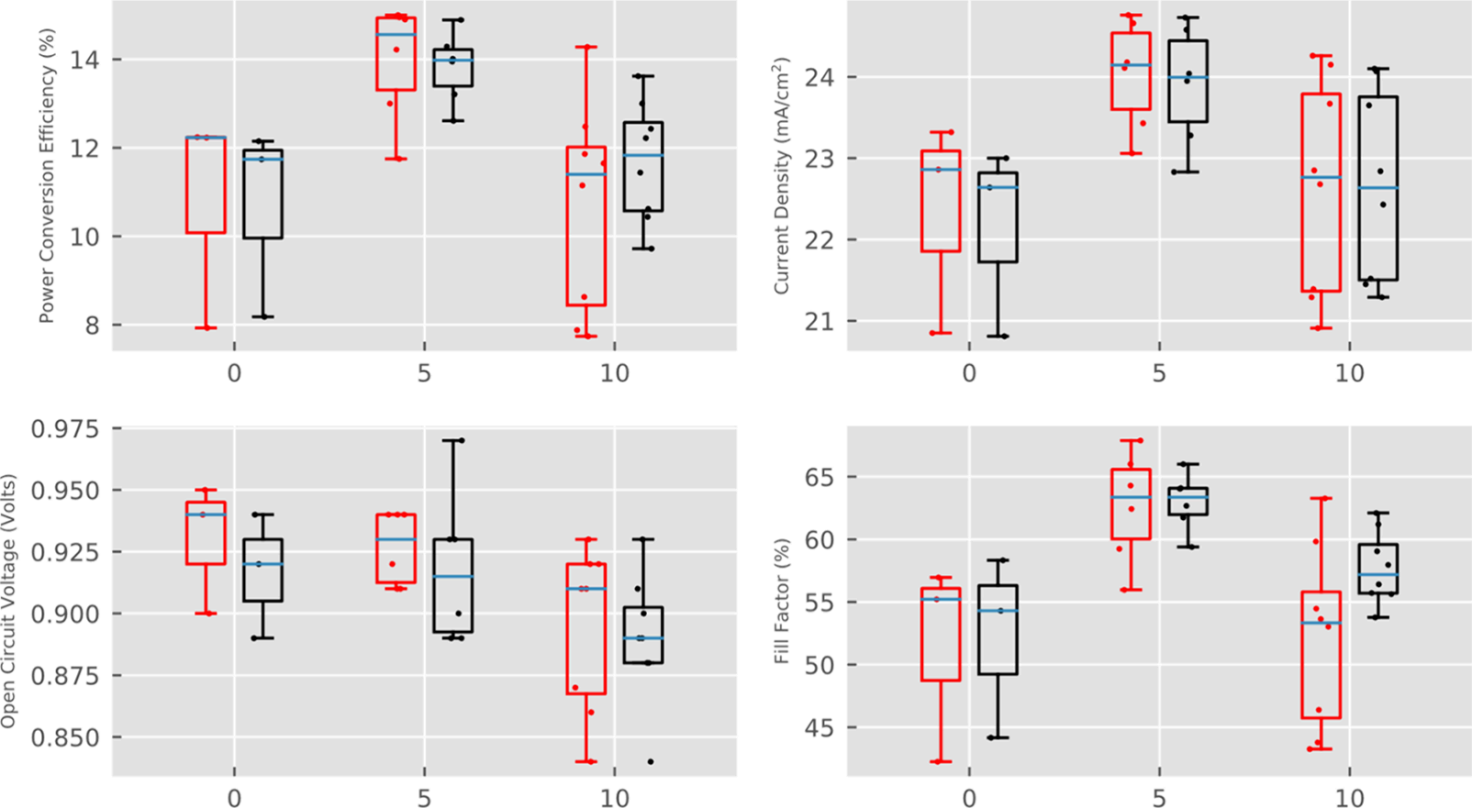
PV parameters of devices made using GEM carbon paste diluted with 0, 5, or 10% of 1-methoxy-2-propanol. Other prints were deposited in the same run, and all were infiltrated at the same time under identical conditions.

Consequently, the 5% devices attained the lowest %UA values of 0.16–0.83% and average *J*_sc_, FF, and PCE values of 24.1 ± 0.7 mA cm^–2^, 62.7 ± 4.4%, and 14.05 ± 1.36%, respectively (Figure 9, Figure S5). In the undiluted samples, %UA values of 0.71–24.00% were obtained, and device FF and *J*_sc_ were much lower. Unexpectedly, despite similar topology and layer thickness, 10% devices were far less reproducible than the 5%, producing %UA values of 0.21 to 14.09% (Figure S5) and PCEs from 8.0 to 14.3% (Figure 9).

It was theorized that higher dilution may provide greater freedom of movement to suspended graphite flakes in the carbon ink, allowing more to settle horizontally to the ZrO_2_ interface, effectively preventing precursor from accessing the underlying mesoporous layers.^16,18^ The amount of horizontal graphite alignment will also depend on environmental conditions such as temperature and air movement during printing and slumping, accounting for the high sample variation.

Cross-sectional analysis of poorly infiltrated 10% samples revealed nearly twice as many horizontally aligned graphite flakes as in the undiluted controls (Figure S6). A 5% dilution therefore provides the optimal balance between reducing filamentation and preventing graphite realignment at the ZrO_2_ interface.

## 4 Conclusions

Optical microscopy and paired image analysis are presented as a fast, scalable, and quantitative method for infiltration comparison. Critically, % uninfiltrated area (%UA) was found to correlate with PCE across multiple batches using different printing regimes. The technique may therefore be suitable for quality control and batch monitoring in future scaled initiatives, where infiltration variation between samples will likely be slight and detailed, nondestructive analysis over large areas is imperative.

This method was then applied to identify key printing issues affecting stack infiltration, namely, mesh marking caused by suboptimal printing regimes. Appearing as distinctive, spaced infiltration defects in the completed device, mesh marking issues were found to cause >6% uninfiltrated area in some cases. The quality of the ZrO_2_ interlayer was found to be particularly important, with even small ZrO_2_ roughness increases capable of causing severe mesh marking in the subsequent carbon print. Identifying these infiltration problems enabled targeted problem solving, namely, adjusting the ZrO_2_ print regime and optimizing carbon pastes. This enabled a 2% PCE increase and significantly improved reproducibility.

## Supporting Information Available

Points of interest during a print pass (Figure S1); photovoltaic parameters (Figures S2 and S3); average thickness and roughness of printed layers (Table S1); optical microscopy images (Figure S4); MO and LUT images of tested device areas (Figure S5); cross-sectional images of complete devices (Figure S6) (PDF)
