# Supplementary material for: Quantifying Infiltration for Quality Control in Printed Mesoscopic Perovskite Solar Cells: A Microscopic Perspective
Source: ACS Appl Energy Mater. 2024 Feb 24;7(5):1938–48. doi: 10.1021/acsaem.3c03056 (PMC10934285; doi:10.1021/acsaem.3c03056)
Supplement: Supplementary file 1 — ae3c03056_si_001.pdf [file ae3c03056_si_001.pdf]

Supporting information

Quantifying Infiltration for Quality Control in Printed Mesoscopic Perovskite Solar Cells: A Microscopic Perspective

Carys A. Worsley,[1] Thomas O. Dunlop,[1] Sarah-Jane Potts,[1] Rodrigo Garcia-Rodriguez,[1] Rebecca S. Bolton,[1] Matthew L. Davies,[1] Eifion Jewell,[1] Trystan M. Watson [1]**

[1] Swansea University, Bay Campus, Skewen, Neath, SA18EN, Wales

* Corresponding authors

c.a.worsley@swansea.ac.uk

t.m.watson@swansea.ac.uk

This file contains supporting data for the above manuscript.

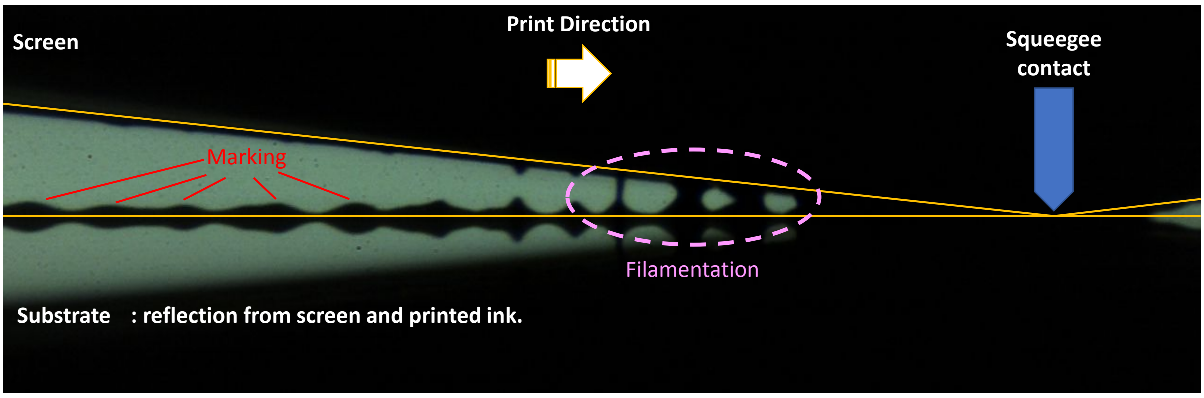

S 1: Labelled image showing points of interest during a print pass during monitoring.

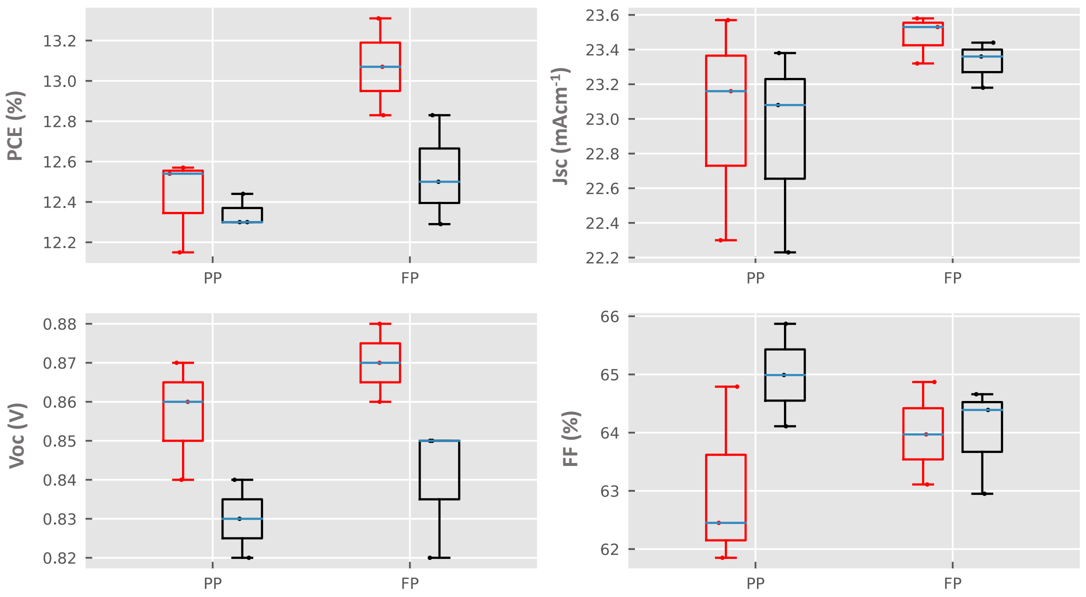

S 2: Photovoltaic parameters of a batch of devices with ZrO_2 layers deposited with two different printing regimes. Three devices in each batch.

Table S 1: Average thickness and roughness of printed layers obtained with an FP method and a fine (130-34) or large (90-48) mesh. Profilometry data were obtained for 3-5 measurements across different prints.

Mesh	Layers	Thickness (mean, µm)	Ra (mean, a.u.)
**Fine (130-34)**	1	1.05 ± 0.09	0.086 ± 0.010
	2	1.94 ± 0.13	0.172 ± 0.006
**Large (90-48)**	1	2.10 ± 0.12	0.131 ± 0.003

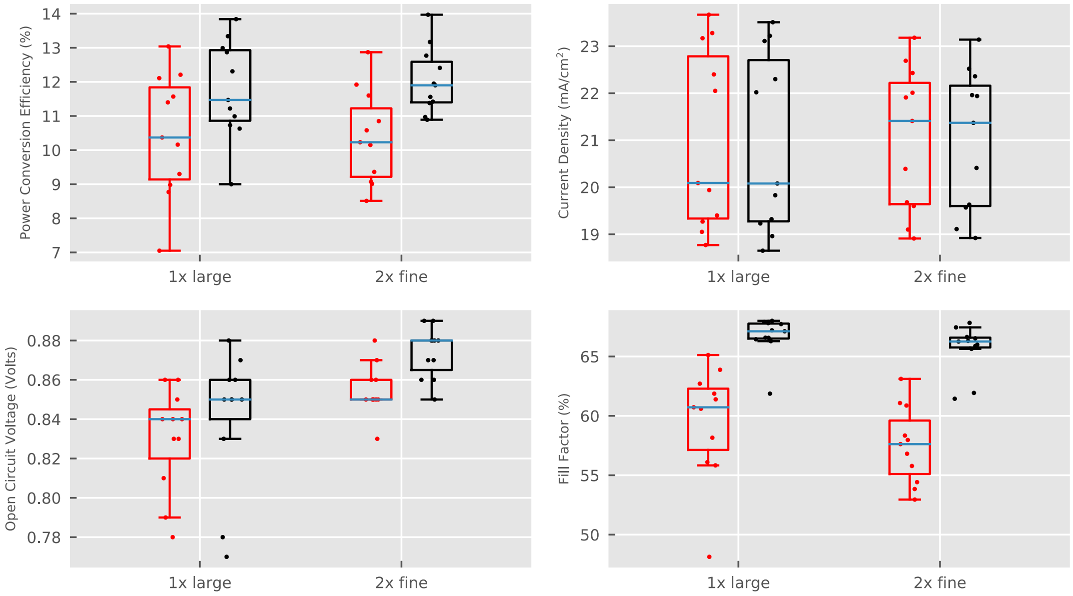

S 3: Photovoltaic parameters of devices produced using a single layer and large mesh (1L) or double layer and fine mesh (1F) for the ZrO_2, printed with the new paste. Eleven 2F and 1L devices each.

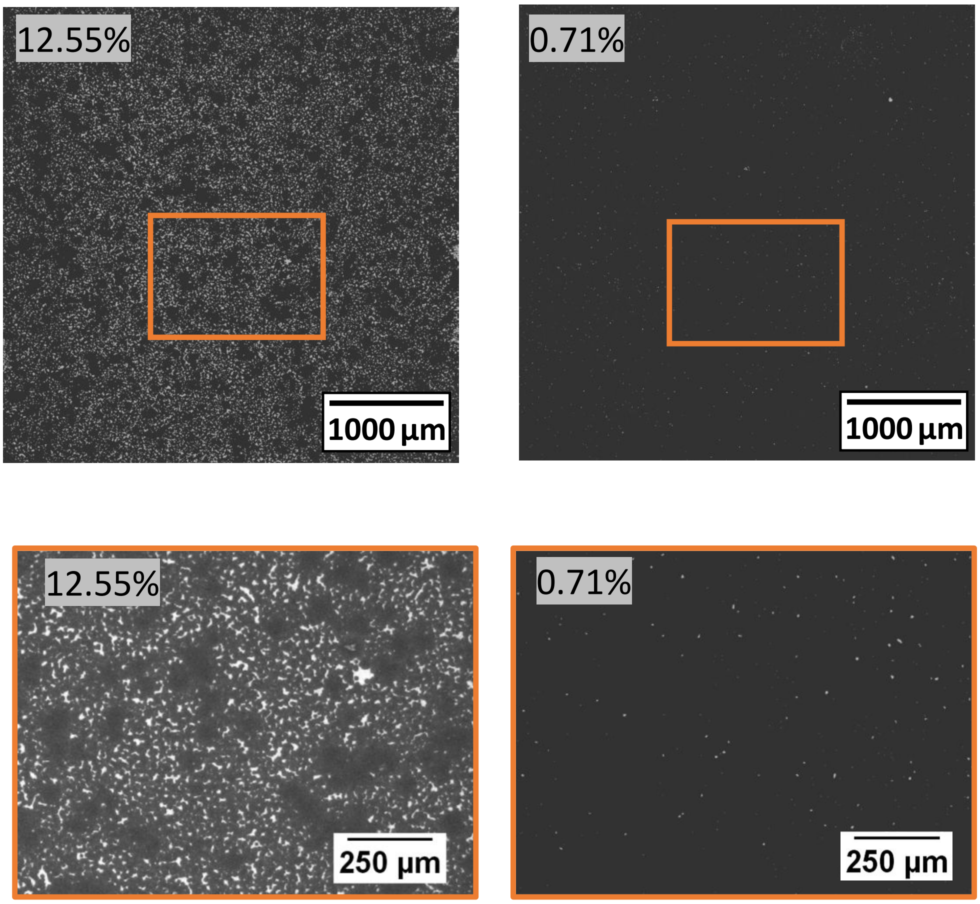

S 4: Optical microscopy images of the varied infiltration observed in FP samples produced with fine mesh ZrO_2 prints. Top images represent the entire active area, bottom images show the orange outlined area at higher magnification. %UA for the entire active area is shown at the top left on each image.

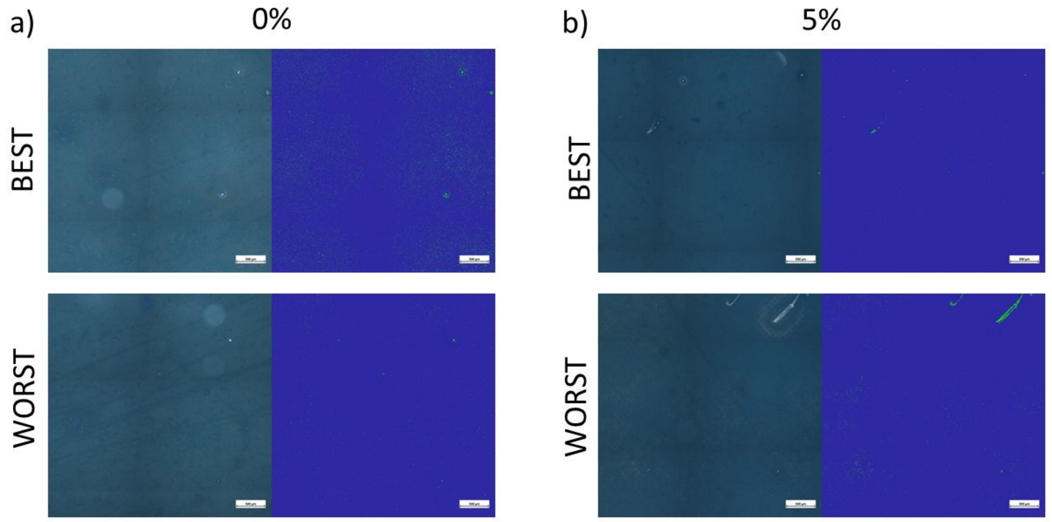

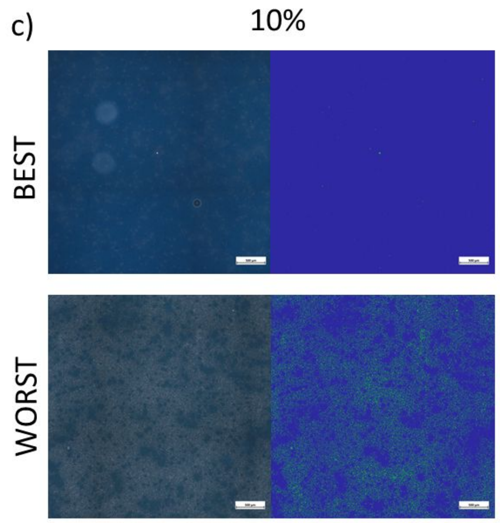

d)

Dilution (%)	Rev PCE (%)	Fwd PCE (%)	% uninfiltrated
0	8.18	7.92	23.99704
0	11.25	12.23	0.70915
0	12.17	12.26	4.176182
5	13.23	13	0.607529
5	14.15	14.6	0.416942
5	13.75	14.08	0.626789
5	14.35	14.98	0.231859
5	12.64	11.76	0.838575
5	14.88	15	0.165325
10	11.45	12.51	0.261444
10	12.24	11.67	0.782811
10	13.66	14.31	0.214747
10	13.02	11.18	1.909559
10	10.62	8.65	8.061834
10	9.75	7.75	12.00626
10	10.44	7.98	14.09391

S5: a)-c) MO and LUT coloured images of tested device areas in cells made using 0, 5 and 10% diluted carbon ink. The best and worst performing device from each set is shown. Table shows forward and reverse PCEs and the calculated uninfiltrated area.

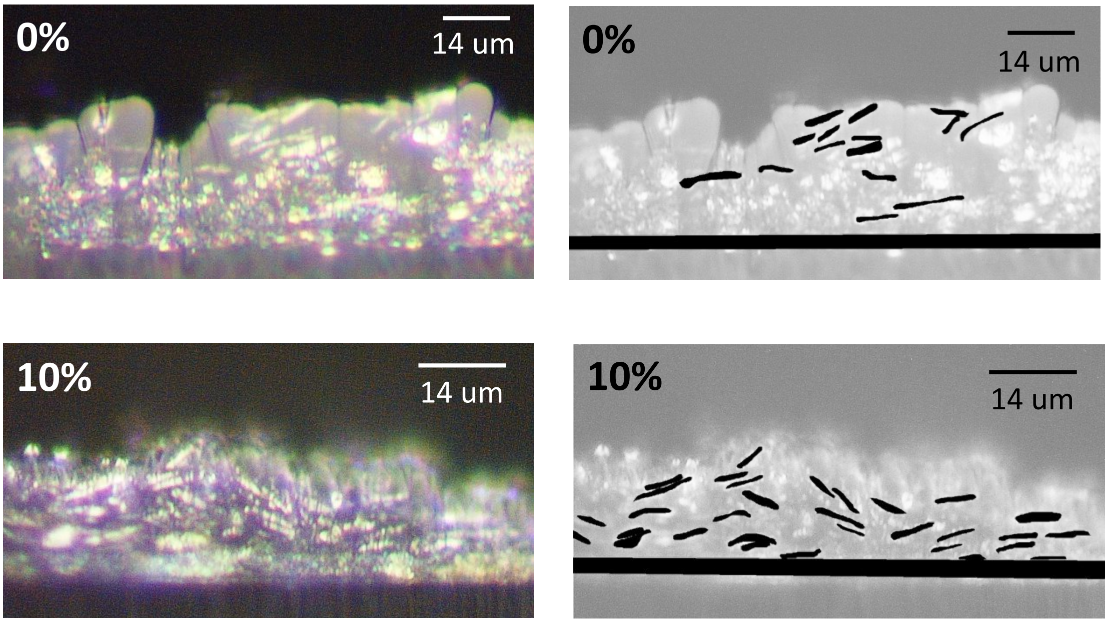

S 6: Cross sectional images of complete devices obtained using an optical microscope and polarising lens to highlight horizontally aligned graphite flakes in the carbon layer. Obvious flakes and the mesoporous TiO_2 and ZrO_2 layers are masked in the RHS images for clarity.
